# Evaluation of a multiplex panel for the diagnosis of acute infectious diarrhea in immunocompromised hematologic patients

**DOI:** 10.1371/journal.pone.0187458

**Published:** 2017-11-03

**Authors:** Izaskun Alejo-Cancho, Francesc Fernández Avilés, Alicia Capón, Cristina Rodríguez, Josep Barrachina, Pilar Salvador, Mª Eugenia Valls, Miriam J. Álvarez-Martínez, Yuliya Zboromyrska, Jordi Vila, Mª Ángeles Marcos

**Affiliations:** 1 Microbiology Department, Hospital Clinic, Barcelona, Spain; 2 Hematology Department, Hospital Clinic, Barcelona, Spain; University of Central Florida College of Medicine, UNITED STATES

## Abstract

**Introduction:**

Diarrhea is a frequent complication in hematologic patients, being an infectious cause frequently suspected. Rapid and accurate detection of gastrointestinal pathogens is vital in immunocompromised hosts. The aim of this study was to compare routine diagnostic methods versus a multiplex polymerase chain reaction (PCR) assay for the diagnosis of infectious diarrhea in immunocompromised hematologic patients.

**Material and methods:**

We conducted a prospective observational study from March 2015 to January 2016 to compare conventional methods for the diagnosis of infectious diarrhea with FIlmArray GI Panel (BioFire-bioMérieux, France). Samples from adult immunocompromised hematologic patients with acute diarrhea were collected. In cases with discordant results, a second multiplex assay was performed (Allplex, Seegene, Korea). The result was considered positive or negative when the same result was obtained by at least two of the methods.

**Results:**

A total of 95 samples were obtained from 95 patients (median age of 52 years (46–64)). Sixty-one (64%) episodes were hospital-acquired and 34 (36%) were community-acquired diarrhea. Twenty-five (26%) patients had a positive microbiological result, being *Clostridium difficile* the most frequent pathogen, followed by *Campylobacter spp* and norovirus. The concordance between FilmArray methods was good (*k* = 0.79). The FilmArray GI panel showed a sensitivity of 95%, a specificity of 100% for positive results. The time required to obtain results was markedly reduced with the use of multiplex PCR methods.

**Conclusions:**

Multiplex molecular panels provide a rapid and sensitive tool for the diagnosis of infectious diarrhea, thereby allowing more timely clinical decisions in immunocompromised hematologic patients.

## Introduction

Diarrhea is a very frequent complication in immunocompromised patients, including hematologic patients. In patients with these underlying conditions diarrhea can be a severe disease, affecting the patient’s quality of life and leading to longer hospitalizations.

Diarrhea can be caused by many different community- and hospital-acquired pathogens, including several bacteria, viruses and, less frequently, parasites. However, the etiology of diarrhea in these patients may be multiple, varying from infectious disease, graft-versus-host disease or drug-induced diarrhea [1]. Even though diarrhea in hematologic patients is a frequent and important issue, there is a lack of studies regarding the etiology of diarrhea in these patients.

Rapid accurate diagnosis of the etiology of diarrhea is required in order to implement the most adequate treatment in immunocompromised patients. Conventional diagnostic techniques, such as culture, microscopy and antigen detection, as well as one-target polymerase chain reaction (PCR) assays, are often laborious and time-consuming, and are only able to detect a limited number of pathogens. Taking these limitations into account faster and more sensitive molecular tests able to simultaneously detect a wide range of bacterial, viral and parasitic pathogens might be helpful in the case of these patients. Several studies have evaluated these assays in various settings, but their performance in hematological patients has yet to be determined [2][3]. The FilmArray GI panel (BioFire-bioMérieux, France) is a FDA-cleared assay that can detect 22 agents of gastroenteritis by a nested multiplex PCR method directly from stool samples, being a very rapid and easy-to-use technique.

The aim of this study was to compare routine diagnostic methods with a multiplex PCR assay (BioFire FilmArray, Gastrointestinal Panel) and to determine the infectious etiology of acute diarrhea in immunocompromised hematologic patients.

## Material and methods

### Study design

This was a prospective observational study carried out from between March 2015 to January 2016. Stool samples were obtained from immunocompromised adult patients (>18 years-old) admitted to the Hematology Department of the Hospital Clinic of Barcelona with acute diarrhea (increased frequency of soft or liquid stools (>3/day) lasting less than 14 days) [4]. Written consent was not obtained from the patients, as the samples were remmants of what had to be taken for other diagnostic purposes and the results would have no impact on the patients. The Ethics Committee of the Hospital Clinic of Barcelona approved the study.

Immunosupression was defined as grades 3–4 neutropenia and/or lymphopenia by the National Cancer Institute Common Toxicity Criteria (NCI-CTC) version 3.0 following the administration of cytotoxic agents or autologous stem-cell transplantation (auto-SCT) or during the first year after allogenic stem-cell transplantation (allo-SCT) [5].

Demographical data including age and sex, and clinical data (community or hospital-acquired diarrhea (acquired >72 hours after admission [4]), diagnosis of graft-versus-host disease, underlying conditions) were obtained from the patients’ clinical history.

A single stool sample was obtained from each patient. The fresh stool specimens were immediately inoculated into different bacterial culture media. The remainder of the sample was stored at 4°C. The remaining routine tests and FilmArray GI Panel were performed only during working hours (i.e. from 8am to 3pm from Monday to Friday). An aliquot of each sample was stored at -20°C for further studies. The maximum time from arrival of the sample until performance of all test was 72 hours. Time and temperature affect especially culture-based procedures, which were performed at the time the sample arrived, so the storage of the samples for further studies should not have an impact on the results of the remaining techniques.

All samples were processed by both routine methods and the FilmArray GI panel. Samples showing a discordant result were tested by an additional method (Allplex (Seegene, Korea)).

### Routine microbiological techniques

All the samples were processed for bacterial, parasitological, viral and *C*. *difficile* toxin study.

The media used for culture of bacteria were as follows: Blood and MacConkey agar plates, SS agar plate (Becton Dickinson, Heidelberg, Germany) for *Shigella* and *Salmonella*, CIN agar plate (Oxoid, Basingstoke, UK) for *Yersinia* and CCDA agar plate (Oxoid) for *Campylobacter* isolation. Additionally, Rappaport-Vassiliadis Salmonella Enrichment Broth (VWK Chemicals, MerckKGaA, Darmstadt, Germany) was used as an enrichment step for the recovery of *Salmonella*, followed by plating onto SS agar. All of the plates were incubated at 37°C a 5% CO_2_ atmosphere for 24–48 hours, except for Campylobacter agar which was incubated in a microaerophilic environment at 42°C.

The final identification of the bacterial isolates was performed by matrix-assisted laser desorption/ionization time-of-flight mass spectrometry (MALDI-TOF (Bruker, Bremen, Germany)). The presence or absence of diarrheagenic *Escherichia coli* was not studied by routine methods in these patients.

*Clostridium difficile* diarrhea was detected following a two-step scheme: detection of GDH by immunochromatography (Health&Research Diagnostics, Spain), followed by PCR (Xpert *C*. *difficile*, Cepheid, California) in the case of a GDH-positive result.

Parasites were detected by microscopy of both, direct and concentrated samples (merthoilate formalin ether method). Modified Kinyoun acid-fast stain was also performed to diagnose *Cryptosporidium* and *Cyclospora cayetanensis*.

The detection of viruses was performed using immunochromatographic tests for rotavirus and adenovirus antigens (VIKIA Rota-adeno, bioMérieux, France) and a real time RT-PCR for detection of norovirus (Xpert Norovirus, Cepheid, California).

Data regarding the time from sample arrival to the laboratory until definitive identification by routine techniques was also obtained. Fresh stool samples were immediately inoculated into different media upon arrival of the samples to the laboratory, but the reading of the plates, microscopic examination, immunochromatographic tests and PCR techniques were only performed during working hours (i.e. from 8am to 3pm from Monday to Friday).

### FilmArray

The FilmArray GI Panel is an automated system in which nucleic acid extraction, amplification and detection occur on a single closed pouch. A nested PCR and melt-curve analysis are performed and analyzed by the FilmArray analyzer. The amount of sample needed is very small and it has a hands-on time of approximately 5 minutes. The total turnaround time is about 1 hour.

All the samples were also processed by the FilmArray GI panel (BioFire-bioMérieux, France) following the manufacturer’s instructions. The FilmArray GI Panel was only performed during working hours. However, clinicians were not informed of the results obtained with this method, and therefore they had not an impact on patient management. This panel includes a total of 22 targets, including bacteria (*Campylobacter jejuni*, *Campylobacter coli*, *Campylobacter upsaliensis*, *Clostridium difficile* (toxin A/B), *Plesiomonas shigelloides*, *Salmonella*, *Yersinia enterocolitica*, *Vibrio parahaemolyticus*, *Vibrio vulnificus*, *Vibrio cholerae* and diarrheagenic *Escherichia coli/Shigella* (Enteroaggregative *E*. *coli* (EAEC), Enteropathogenic *E*. *coli* (EPEC), Enterotoxigenic *E*. *coli* (ETEC) lt/st, Shiga-like toxin-producing *E*. *coli* (STEC) stx1/stx-2, *E*. *coli* O157, *Shigella*/Enteroinvasive *E*. *coli* (EIEC)), parasites (*Cryptosporidium*, *Cyclospora cayetanensis*, *Entamoeba histolytica*, *Giardia lamblia*) and viruses (Adenovirus F 40/41, Astrovirus, norovirus GI/GII, Rotavirus A, Sapovirus (I, II, IV and V)). The detection methods used for each of the pathogens are further explained in the panel insert (https://www.online-ifu.com/ITI0030).

### Confirmation testing

Discordant results between routine methods and the FilmArray GI Panel were confirmed using a second multiplex PCR panel, Allplex (Seegene, Korea), following the manufacturer’s instructions. This assay is made up of 4 different panels that include the following: Panel 1 Virus (Norovirus GI and GII, Rotavirus, Adenovirus, Astrovirus, and Sapovirus), Panel 2 Bacteria I (*Campylobacter spp*, *Clostridium difficile* toxin B, *Salmonella spp*, EIEC/*Shigella spp*, *Vibrio spp*, *Yersinia enterocolitica*, *Aeromonas spp*), Panel 3 Bacteria II (*Clostridium difficile* hypervirulent, *E*. *coli* O157, EHEC, EPEC, ETEC, and EAEC) and Panel 4 Parasite (*Giardia lamblia*, *Entamoeba histolytica*, *Cryptosporidium spp*, *Blastocystis hominis*, *Dientamoeba fragilis*, and *Cyclospora cayetanensis*).

The stool specimen was previously shaken in a bead tube with lysis buffer and then centrifuged. Then 200 μl of the supernatant were used for automatic extraction with the EZ1 Virus Mini Kit (Qiagen), obtaining the nucleic acids in 60 μl of AVE elution buffer.

### Result interpretation

A result was considered positive or negative when the same result was obtained by at least two of the three methods.

### Statistical analysis

We show the number and percentage of patients for categorical variables and the median (interquartile range) for continuous variables. The Cohen’s kappa coefficient (*k*) [6] was calculated to measure the agreement between the FilmArray GI Panel and the routine methods. The sensitivity, specificity, positive predictive value (PPV), negative predictive value (NPV) and likelihood ratios (LR) of positive detection of the FilmArray GI Panel and routine methods were also calculated for comparison with the final diagnosis. All tests were 2-tailed and significance was set at 0.005. All analyses were performed with IBM SPSS Statistics 22.0 (Armonk, New York, USA) and Epidat 3.1 (Santiago de Compostela, A Coruña, Spain).

### Ethical aspects

This study was approved by the Ethics Committee of the Hospital Clinic of Barcelona, which considered that written consent was not necessary, as the samples had to be taken for other diagnostic purposes and the results had no impact on the patients.

BioMerieux España S.A.U. provided the kits of FilmArray used in this study. They did not, however, take part in designing the study or in analyzing the results.

## Results

### Patients and samples

During the study period, a total of 95 stool samples were obtained from 95 patients with a median age of 52 years (46–64). Forty-one (43%) patients were within the first year after an allo-SCT, and the remaining patients were immunocompromised after an auto-SCT (n = 19; 20%) or secondary to chemotherapy for acute leukemia (n = 19; 20%), lymphoma (n = 9; 10%), multiple myeloma (n = 3; 3%), myelodysplastic syndrome (n = 2; 2%) or other underlying immunosuppressive conditions (n = 2; 2%). Graft-versus-host disease was suspected in 5 patients with allogenic bone marrow transplantation, but it was confirmed in only two of these patients.

Regarding the acquisition of diarrhea, 61 episodes were classified as nosocomial diarrhea (64%) and 34 were community-acquired diarrhea (36%).

### Laboratory results

A positive definite diagnosis was achieved in 25 patients (26%), with the same pathogen being detected in at least two of the three methods tested. Table 1 shows the positive results obtained with all the methods and the final interpretation of them. The percentage of positive samples excluding those with only diarrheagenic *E*.*coli* was 22%.

**Table 1:** Microbiological results obtained by each method and the final diagnosis of the positive cases.

Patient	Diagnostic methods			Final Diagnosis
	FilmArray	Routine	Allplex	
7	*Clostridium difficile* toxin A/B	*Clostridium difficile* toxin A/B	NA	*Clostridium difficile* toxin A/B
12	*Campylobacter spp*	*Campylobacter jejuni*	NA	*Campylobacter spp*
18	Rotavirus	Rotavirus	NA	Rotavirus
20	*Campylobacter spp* + *Clostridium difficile* toxin A/B	*Campylobacter jejuni* + *Clostridium difficile* toxin A/B	NA	*Campylobacter spp* + *Clostridium difficile* toxin A/B
21	EPEC + *Clostridium difficile* toxin A/B	Negative	EPEC + *Clostridium difficile* toxin A/B	EPEC + *Clostridium difficile* toxin A/B
23	*Clostridium difficile* toxin A/B	*Clostridium difficile* toxin A/B	NA	*Clostridium difficile* toxin A/B
24	EPEC + ETEC	Negative	EPEC + ETEC	EPEC + ETEC
27	EPEC	Negative	EPEC	EPEC
28	EPEC	Negative	EPEC	EPEC
44	EPEC + Norovirus	Adenovirus + Norovirus	EPEC + Norovirus	EPEC + Norovirus
47	*Clostridium difficile* toxin A/B	*Clostridium difficile* toxin A/B	NA	*Clostridium difficile* toxin A/B
48	EPEC	Negative	Negative	Negative
55	*Clostridium difficile* toxin A/B	*Clostridium difficile* toxin A/B	NA	*Clostridium difficile* toxin A/B
56	Norovirus	Norovirus	NA	Norovirus
58	ETEC + EPEC	Adenovirus	ETEC + Adenovirus	ETEC + Adenovirus
60	EAEC + *Campylobacter spp*	Negative	EAEC + *Campylobacter spp*	EAEC + *Campylobacter spp*
70	EPEC	Negative	Negative	Negative
73	*Clostridium difficile* toxin A/B	*Clostridium difficile* toxin A/B	NA	*Clostridium difficile* toxin A/B
76	*Campylobacter spp* + EPEC	*Campylobacter jejuni*	*Campylobacter spp* + EPEC	*Campylobacter spp* + EPEC
79	*Campylobacter spp*	Negative	*Campylobacter spp*	*Campylobacter spp*
84	*Salmonella spp* + EPEC + Norovirus	*Salmonella typhimurium* + Norovirus	*Salmonella spp* + EPEC + Norovirus	*Salmonella spp* + EPEC + Norovirus
85	Norovirus	Norovirus	NA	Norovirus
86	*Campylobacter spp* + Giardia lamblia	*Campylobacter jejuni*	*Campylobacter spp* + Giardia lamblia	*Campylobacter spp* + Giardia lamblia
90	*Clostridium difficile* toxin A/B + Norovirus	Norovirus	*Clostridium difficile* toxin A/B + Norovirus	*Clostridium difficile* toxin A/B + Norovirus
92	EPEC	Negative	EPEC	EPEC
97	*Clostridium difficile* toxin A/B	*Clostridium difficile* toxin A/B	NA	*Clostridium difficile* toxin A/B
100	*Clostridium difficile* toxin A/B	*Clostridium difficile* toxin A/B	NA	*Clostridium difficile* toxin A/B

Abbreviations: EAEC Enteroaggregative *E*. *coli*, EPEC Enteropathogenic *E*. *coli*, ETEC Enterotoxigenic *E*. *coli* lt/st, NA not applicable

A total of 70 samples were negative by both methods, ruling out infectious etiology in this group of patients.

Diarrheagenic *E*. *coli* were detected by the FilmArray GI Panel, but these pathogens were not searched for by routine methods. Diarrheagenic *E*.*coli* were detected in 12 samples using the Filmarray GI Panel, 10 of which were confirmed by Allplex. *E*. *coli* was the only pathogen detected in 4 of these samples.

Excluding diarrheagenic *E*. *coli*, the most frequent etiological agent was *C*. *difficile*, being identified in 10 samples (in two samples the immunochromatographic test was negative, and thus PCR was not performed, leading to routine methods not detecting *C*. *difficile*). The second most frequent pathogen was *Campylobacter spp*, being detected in 6 samples (not detected by culture techniques in 2 cases). The third most frequent pathogen was norovirus which was detected in 5 cases. Other less frequent etiologies were salmonella, adenovirus, rotavirus and giardia. Table 2 shows the frequency of the pathogens detected in total, by each method, their frequency in coinfection and the type of acquisition.

**Table 2:** Summary of the pathogens detected by routine methods and FilmArray, the percentage of coinfection and the acquisition.

Pathogen	Total (%)	Coinfection (%)	Detected by routine	Detected by FilmArray	Acquisition	
					Nosocomial	Community
**Bacteria**						
*C*. *difficile*	10 (27.78)	3 (30)	8	10	5	5
*Campylobacter spp*	6 (16.67)	4 (66.67)	4	6	2	4
*Salmonella spp*	1 (2.78)	1 (100)	1	1		1
*EAEC*	1 (2.78)	1 (100)	NA	1		1
*EPEC*	8 (22.22)	5 (62.5)	NA	11	1	7
*ETEC*	2 (5.56)	2 (100)	NA	2	1	1
**Virus**						
*Adenovirus*	1 (2.78)	1 (100)	1	0	1	
*Norovirus*	5 (13.89)	3 (60)	5	5	3	2
*Rotavirus*	1 (2.78)	0	1	1		1
**Parasites**						
*G*. *lamblia*	1 (2.78)	1 (100)	0	1	1	

Abbreviations: EAEC Enteroaggregative *E*. *coli*, EPEC Enteropathogenic *E*. *coli*, ETEC Enterotoxigenic *E*. *coli* lt/st

In 15 samples only one pathogen was found, whereas 10 patients presented coinfections (9 coinfections with 2 pathogens and 1 coinfection with 3 pathogens).

Among the samples with a positive microbiologic result, there were 10 hospital-acquired (40%) and 15 community-acquired diarrhea (60%). In the 10 cases of nosocomial diarrhea the most frequent pathogen was also *C*. *difficile* in 5 cases (50%) followed by norovirus in 3 (30%).

The concordance between the FilmArray GI Panel and routine methods was good (*k* = 0.79, 95% CI [0.65 to 0.91], p<0.001). The positive results obtained using the FilmArray GI Panel had a sensitivity of 95%, a specificity of 100%, a PPV of 100%, a NPV of 99%, a LR+ of 140 and a LR- of 0.05, being 75%, 99%, 94%, 94%, 57 and 0.25, respectively with routine methods (Table 3). As mentioned previously, routine methods did not take diarrheagenic *E*. *coli* into account.

**Table 3:** Diagnostic accuracy of positive detection of FilmArray and Routine methods.

Results	FilmArray		Routine methods	
	Value	95% CI	Value	95% CI
Sensitivity (%)	95.2	83.8 to 100.0	75.0	53.5 to 96.5
Specificity (%)	100.0	99.3 to 100.0	98.7	95.5 to 100.0
PPV (%)	100.0	97.5 to 100.0	93.8	78.8 to 100.0
NPV (%)	98.7	95.4 to 100.0	93.8	87.8 to 99.7
LR+	140.0^a^	8.8 to 2219.0^a^	57.0	8.0 to 406.0
LR-	0.05	0.01 to 0.32	0.25	0.12 to 0.54

Abbreviations: CI confidence interval; LR, likelihood ratio; NPV, negative predictive value; PPV, positive predictive value.

^a^ 0.5 added to cells to estimate LR+ and CI's of the LR+.

Regarding the time to achieving the results, the routine methods took up to 89 hours (53–110) for a definite result.

The samples from all 5 patients with suspected graft-versus-host disease were negative with both the routine methods and the FilmArray GI Panel.

## Discussion

The etiology of the diarrhea in our cohort of immunocompromised hematologic patients was infectious in 26% of the cases. This percentage of infectious diarrhea is similar to that reported in previous studies focusing in other populations of immunocompromised patients [7] and highlights the importance of an infectious etiology in this group of patients.

The most frequently detected pathogen was *C*. *difficile* which is in agreement with previous studies [8][9], showing that this etiology of diarrhea should be taken into account in these patients, especially if they have been hospitalized for a prolonged period of time. However, even though until recently this pathogen was considered a nosocomial pathogen, we found that half of our cases were not hospital-acquired. Again, this is in agreement with a population-based study that found that 41% of *C*. *difficile* infections were community-acquired [10]. In view of the present data, the lack of previous hospitalization or contact with other health care settings should not exclude the suspicion of *C*. *difficile* diarrhea. However, the classification of episodes of diarrhea as hospital- or community-acquired in immunocompromised patients is not easy, since these patients are frequently in contact with the health care system and often require hospitalization. In addition, according to our algorithm, two cases were not detected because of a negative GDH result. When The FilmArray results were available, these samples were re-tested by RT-PCR and positive results were obtained. This shows that our diagnostic algorithm could miss cases with false negative immunochromatography results which would not remain unnoticed with the FilmArray GI Panel.

*Campylobacter* were the second most frequently detected bacteria in our study. In 2 out of the 6 cases, the microorganism was not isolated in the culture. The lack of sensitivity of culture techniques for *Campylobacter* has been reported previously [11], although this has improved greatly with the new molecular techniques.

The most frequent viral pathogen was Norovirus. Several studies have reported that immunosupressed patients seem to be more prone to norovirus infection than other patients [12]. This pathogen is also considered an important cause of nosocomial diarrhea, although we found that nearly half of our cases were community-acquired.

An interesting finding of this study is the high number of diarrheagenic *E*. *coli* detected (10 cases, being the only pathogen found in 4), suggesting the involvement of this microorganism as a cause of diarrhea in immunocompromised patients. These pathogens have also been described in diarrhea in other settings, such as travelers returning from developing countries [13]. Several studies have also described the importance of diarrheagenic *E*. *coli* as a causative agent of diarrhea in children in developed countries[14]. Indeed, the use of multiplex panels for the etiologic study of diarrhea has shown that this previously unsuspected pathogen is present in many patients’ stool samples [15][16]. However, further studies are needed to analyze the role of these pathogens in immunocompromised hematologic patients. In our study EPEC was the most frequent diarrheagenic *E*. *coli* detected. Some studies have shown that this pathogen is frequently found in asymptomatic patients and healthy carriers [17][18], thereby underlining the importance of detecting this pathogen in these patients.

In 70 patients, who were also immunocompromised patients admitted to the Hematology Department with acute diarrhea, negative results were obtained by both methods, ruling out infectious diarrhea. This is also an important finding, as it would allow the clinician to consider other possible non-infectious etiologies (such as drug-induced diarrhea or graft-versus-host disease) which are frequent in these patients.

Regarding the diagnostic methods evaluated in this study, the FilmArray GI Panel showed good concordance with the routine methods and increased sensitivity compared with these latter methods. The FilmArray GI Panel also allowed the diagnosis of unsuspected pathogens such as diarrheagenic E. coli or even parasites and improved the number of coinfections detected.

The ease of use of the FilmArray GI Panel allows non-trained personal to perform the analysis 24 hours a day, 7 days a week providing results in less than one hour, compared to the longer time-to-results obtained with routine methodology. The speed in obtaining results using multiplex testing is important not only when positive results are obtained, but also in order to rule out an infectious etiology after performing a sensitive test that includes the most frequent pathogens. A negative result in immunocompromised hematologic patients would suggest that the episode could be due to non-infectious causes such as immunosuppressive drugs or graft-versus-host disease. On the other hand, a rapid diagnostic tool can have a great impact on the control of nosocomial infections allowing the implementation of timely measures to prevent the spread of intection and avoid the development of new cases.

Multiplex assays do, however, present some limitations. Many bacterial infections require bacterial culture, as isolation is needed in order to perform susceptibility testing. In addition, a higher sensitivity could imply an increase in the detection of non-viable pathogens or pathogens that are actually colonizers without causing infection [19]. Moreover, the FilmArray GI Panel represents a considerable increase in cost over routine techniques. However, evaluation of cost-effectiveness should be further studied [20], taking into account not only reagents, but also technician time, as weel as instrumentations and hospital-associated fees (e.g. isolation days and treatment decisions). Indeed, targeted use of the FilmArray GI Panel in specific populations could have an impact on treatment costs and also on costs derived from nosocomial outbreaks.

This study presents some limitations including the reduced number of patients and that its descriptive nature which has no impact on patient management. However, the results of this study showed that infectious agents cause a considerable number of diarrheal episodes in immunosupressed hematological patients. These patients are a critical population that could benefit from a rapid and sensitive diagnosis in which the confirmation of a positive result is as important as ruling it out, since the treatment may substantially differ. The use of multiplex PCRs may significantly reduce the time between sample reception and result reporting to the clinicians, and it may help them to correctly classify the cause of the diarrhea and implement the most adequate therapy. A positive result could allow the immediate start of antibiotic therapy, which could be later adjusted after antimicrobial susceptibility testing is performed by other methods. A negative result could reduce the amount of antibiotics prescribed, improving patient management and reducing the appearance of new resistant bacteria. However, this was only an observational study and these aspects were not evaluated, needing further studies to correctly assess the effectiveness of these new diagnostic approaches.

## References

[1] Krones E, Högenauer C. Diarrhea in the Immunocompromised Patient. Gastroenterol Clin North Am. 2012;41: 677–701. doi: 10.1016/j.gtc.2012.06.009 22917171 10.1016/j.gtc.2012.06.009

[2] Deng J, Luo X, Wang R, Jiang L, Ding X, Hao W, et al A comparison of Luminex xTAG® Gastrointestinal Pathogen Panel (xTAG GPP) and routine tests for the detection of enteropathogens circulating in Southern China. Diagn Microbiol Infect Dis. Elsevier Inc.; 2015;83: 325–330. doi: 10.1016/j.diagmicrobio.2015.07.024 26318973 10.1016/j.diagmicrobio.2015.07.024

[3] Gu Z, Zhu H, Rodriguez A, Mhaissen M, Schultz-Cherry S, Adderson E, et al Comparative Evaluation of Broad-Panel PCR Assays for the Detection of Gastrointestinal Pathogens in Pediatric Oncology Patients. J Mol Diagnostics. American Society for Investigative Pathology and the Association for Molecular Pathology; 2015;17: 715–721. doi: 10.1016/j.jmoldx.2015.06.003 26321042 10.1016/j.jmoldx.2015.06.003

[4] Guerrant RL, Gilder T Van, Steiner TS, Thielman NM, Slutsker L, Tauxe R V, et al Practice Guidelines for the Management of Infectious Diarrhea. Clin Infect Dis. 2001;32: 33150 doi: 10.1086/318514 11170940 10.1086/318514

[5] National Institute of Cancer. Common Terminology Criteria for Adverse Events (CTCAE). NIH Publ. 2010;2009: 0–71. doi: 10.1080/00140139.2010.489653

[6] Landis JR, Koch GG. The Measurement of Observer Agreement for Categorical Data. Biometrics. 1977 3;33: 159–174. doi: 10.2307/2529310 843571

[7] Polage CR. Microbiologic Testing in Post–Solid Organ Transplant Diarrhea. Clin Infect Dis 2014; 1–3. doi: 10.1093/cid/ciu884 10.1093/cid/ciu884 PMC4329923 25371493

[8] Spadão F, Gerhardt J, Guimarães T, Dulley F, Almeida Junior JN De, Batista MV, et al Incidende of diarrhea by Clostridium difficile in hematologic patients and hematopoietic stem cell transplantation patients: risk factors for severe forms and death. Rev Inst Med Trop Sao Paulo. 2014;56: 325–331. doi: 10.1590/S0036-46652014000400010 25076434 10.1590/S0036-46652014000400010 PMC4131819

[9] Bobak D, Arfons LM, Creger RJ, Lazarus HM. Clostridium difficile-associated disease in human stem cell transplant recipients: coming epidemic or false alarm? Bone Marrow Transplant. 2008;42: 705–713. doi: 10.1038/bmt.2008.317 18836490 10.1038/bmt.2008.317

[10] Khanna S, Pardi DS, Aronson SL, Patricia P, Orenstein R, Sauver JLS, et al The epidemiology of community-acquired Clostridium difficile infection: A population-based study. Am J Gastroenterol 2012: 89–95. doi: 10.1038/ajg.2011.398 22108454 10.1038/ajg.2011.398 PMC3273904

[11] Bessde E, Delcamp A, Sifr E, Buissonnire A, Mgraud F. New methods for detection of campylobacters in stool samples in comparison to culture. J Clin Microbiol. 2011;49: 941–944. doi: 10.1128/JCM.01489-10 21209172 10.1128/JCM.01489-10 PMC3067684

[12] Doshi M, Woodwell S, Kelleher K, Mangan K, Axelrod P. An outbreak of norovirus infection in a bone marrow transplant unit. Am J Infect Control. Elsevier Inc; 2013;41: 820–823. doi: 10.1016/j.ajic.2012.10.025 23415769 10.1016/j.ajic.2012.10.025

[13] Vargas M, Gascón J, Gallardo F, Jimenez De Anta MT, Vila J. Prevalence of diarrheagenic Escherichia coli strains detected by PCR in patients with travelers’ diarrhea. Clin Microbiol Infect. 1998;4: 682–688. doi: 10.1111/j.1469-0691.1998.tb00652.x 11864275 10.1111/j.1469-0691.1998.tb00652.x

[14] Amisano G, Fornasero S, Migliaretti G, Caramello S, Tarasco V, Savino F. Diarrheagenic escherichia coli in acute gastroenteritis in infants in north-west italy. New Microbiol. 2011;34: 45–51. 21344146

[15] De Rauw K, Detemmerman L, Breynaert J, Pirard D. Detection of Shiga toxin-producing and other diarrheagenic Escherichia coli by the BioFire FilmArray Gastrointestinal Panel in human fecal samples. Eur J Clin Microbiol Infect Dis. European Journal of Clinical Microbiology & Infectious Diseases; 2016;35: 1479–1486. doi: 10.1007/s10096-016-2688-7 27259710 10.1007/s10096-016-2688-7

[16] Coste JF, Vuiblet V, Moustapha B, Bouin A, Lavaud S, Toupance O, et al Microbiological diagnosis of severe diarrhea in kidney transplant recipients by use of multiplex PCR assays. J Clin Microbiol. 2013;51: 1841–1849. doi: 10.1128/JCM.03366-12 23554205 10.1128/JCM.03366-12 PMC3716061

[17] Platts-Mills JA, Gratz J, Mduma E, Svensen E, Amour C, Liu J, et al Association between stool enteropathogen quantity and disease in Tanzanian children using TaqMan Array Cards: A nested case-control study. Am J Trop Med Hyg. 2014;90: 133–138. doi: 10.4269/ajtmh.13-0439 24189366 10.4269/ajtmh.13-0439 PMC3886409

[18] Enserink R, Scholts R, Bruijning-Verhagen P, Duizer E, Vennema H, De Boer R, et al High detection rates of enteropathogens in asymptomatic children attending day care. PLoS One. 2014;9(2):e89496 doi: 10.1371/journal.pone.0089496 24586825 10.1371/journal.pone.0089496 PMC3933542

[19] Zboromyrska Y, Vila J. Advanced PCR-based molecular diagnosis of gastrointestinal infections: challenges and opportunities. Expert Rev Mol Diagn. Taylor & Francis; 2016;16: 631–640. doi: 10.1586/14737159.2016.1167599 26986537 10.1586/14737159.2016.1167599

[20] Binnicker MJ. Multiplex Molecular Panels for Diagnosis of Gastrointestinal Infection: Performance, Result Interpretation, and Cost-Effectiveness. J Clin Microbiol 2015; 53:3723–3728. doi: 10.1128/JCM.02103-15 26311866 10.1128/JCM.02103-15 PMC4652086

